# Systematic review of the status of *pfhrp2* and *pfhrp3* gene deletion, approaches and methods used for its estimation and reporting in *Plasmodium falciparum* populations in Africa: review of published studies 2010–2019

**DOI:** 10.1186/s12936-019-2987-4

**Published:** 2019-11-06

**Authors:** Bosco B. Agaba, Adoke Yeka, Sam Nsobya, Emmanuel Arinaitwe, Joaniter Nankabirwa, Jimmy Opigo, Paul Mbaka, Chae Seung Lim, Joan N. Kalyango, Charles Karamagi, Moses R. Kamya

**Affiliations:** 1 School of Medicine, College of Health Sciences Makerere University, Kampala, Uganda; 2 Clinical Epidemiology Unit, Makerere University Kampala, Kampala, Uganda; 3 National Malaria Control Programme, Kampala, Uganda; 4 School of Public Health, Makerere University College of Health Sciences, Kampala, Uganda; 5 School of Biomedical Sciences, Makerere University College of Health Sciences, Kampala, Uganda; 6 Infectious Diseases Research Collaboration, Kampala, Uganda; 7 World Health Organization Country Office, Kampala, Uganda; 8 Department of Laboratory Medicine, College of Health Sciences, Korea University, Seoul, South Korea

**Keywords:** Malaria rapid diagnostic tests, *Plasmodium falciparum*, Histidine rich protein 2 gene, Systematic review, Histidine rich protein 3, Gene deletion, Africa

## Abstract

**Background:**

Malaria rapid diagnostic tests based on histidine-rich protein-2 have played a vital role in improving malaria case management and surveillance particularly in Africa, where *Plasmodium falciparum* is predominant. However, their usefulness has been threatened by the emergence of gene deletion on *P. falciparum histidine rich protein 2* (*pfhrp2*) and *P. falciparum histidine rich protein 3* (*pfhrp3*). Use of standard and recommended methods is key for accurate investigation, confirmation and reporting of *pfhrp2* and *pfhrp3* gene deletion.

**Methods:**

A systematic review was conducted to assess the status, methods and approaches that have been used for investigation, confirmation and reporting of *pfhrp2* and *pfhrp3* gene deletion in Africa. An online search was done using PubMed and MEDLINE Google Scholar for all articles published in English on *pfhrp2/3* gene deletion in Africa. Relevant articles that met the inclusion criteria were summarized and assessed based on the protocol recommended by the World Health Organization for confirmation and reporting of *pfhrp2/3* gene deletion.

**Results:**

The search identified a total of 18 articles out of which 14 (77.7%) fulfilled the criteria for inclusion and were retained for review. The articles were distributed across 12 countries where the p*fhrp2* and *pfhrp3* gene deletion studies were conducted and reported. The level of *pfhrp2/3* gene deletion across selected studies in Africa ranged from the highest 62% to the lowest 0.4%. There was wide variation in methods and approaches including study designs, size and sampling and whether both *pfhrp2* and *pfhrp3* double deletions or *pfhrp2* single deletion were investigated, with a wide variation in laboratory methods.

**Conclusion:**

Based on the review, there is evidence of the presence of *pfhrp2/3* gene-deleted *P. falciparum* parasites in Africa. The approaches and methods used for investigation, confirmation and reporting of *pfhrp2/3* deleted parasites have varied between studies and across countries. Countries that are considering plans to investigate, confirm and report *pfhrp2/3* deletion should use recommended standard and harmonized methods to prevent unnecessary recommendations for costly switch of RDTs in Africa.

## Background

The World Health Organization (WHO) estimated that there were 219 million cases of malaria and 435,000 malaria deaths and nearly half of the world’s population was at risk of malaria infection in 2017 [1, 2]. The WHO African Region continues to carry a disproportionately high share of the global malaria burden contributing 92% (200 million) malaria cases and 91% of malaria deaths. *Plasmodium falciparum* is the most prevalent malaria species in the WHO African region, accounting for 99.7% of estimated malaria cases in 2017 [1, 2].

Efforts to reduce the burden of malaria in Africa have mostly included the use of long-lasting insecticide-treated nets (LLINs), indoor residual spraying (IRS) with insecticides, intermittent preventive therapy (IPT), diagnosis and treatment. Case management which involves testing and treatment with artemisinin-based combination therapy (ACT) is a major intervention for malaria control [1, 2]. The WHO recommends parasitological confirmation of malaria in all suspected cases prior to treatment with ACT. Nearly all countries in Africa adopted this as policy and have shifted from clinical to parasite-based diagnosis with microscopy or rapid diagnostic tests (RDTs) [1–3]. Due to systemic challenges associated with blood smear microscopy, RDTs are becoming increasingly the most used method to test for malaria among suspected malaria patients in sub-Saharan Africa [1, 2]. In 2017 alone, an estimated 75% of malaria tests were conducted using RDTs, up from 40% in 2010 and an estimated 276 million rapid diagnostic tests (RDTs) were sold globally [1, 2]. Due to the dominance of *P. falciparum*, over 90% of RDTs used for the diagnosis of malaria in sub-Saharan Africa are HRP2-based [1, 2]. *Plasmodium falciparum* specific RDTs specifically recognize HRP2 antigen that encodes for the *pfhrp2* gene and whose antibodies cross-react with histidine-rich protein 3 (*pfhrp3*) antibodies due to high degree of similarity in amino acid sequence [3–5]. However, recent publications have indicated that a substantial number of malaria parasites in the Amazon region and some parts of Africa and Asia are lacking the *pfhrp2* and *pfhrp3* genes. *Plasmodium falciparum* parasites lacking the *pfhrp2/3* gene do not express HRP2 protein antigen threatening the usefulness of HRP2 RDTs in malaria diagnosis [3, 4, 6]. The first *P. falciparum* parasites with *pfhrp2* and *pfhrp3* gene deletions were reported in the Amazon basin in 2010 by Gamboa et al. [4]. However recent evaluations of malaria parasites revealed the presence of *pfhrp2/3* gene deletions outside the Amazon region in Africa and India [6]. The occurrence of *P. falciparum* with missing *pfhrp2/3* genes pose a public health threat as a large number of malaria infected patients will go undetected by the HRP2 RDTs and, therefore, remain untreated leading to increased risk of malaria morbidity and mortality, and continued malaria transmission [3, 5, 6].

The WHO recommends a policy switch to more effective alternative non-HRP2 RDTs, when the prevalence of *pfhrp2*-deleted parasites meets or exceeds the lower 90% confidence interval for 5% prevalence, or a plan for change over a longer time frame if deletions are present but < 5% [7]. In Africa, a number of studies have reported occurrence of *pfhrp2* and *pfhrp3* gene deletions [8–18]. Due to the high prevalence of *pfhrp2/3* gene deletion, countries, such as Eritrea have introduced non-HRP2 alternative RDTs that are able to detect gene-deleted parasites [11]. However, the costs and resources associated with the switch of national malaria diagnostic strategies from HRP2 to alternative non-HRP2 based RDTs are enormous. In addition to the costs associated with training, non-HRP2 based RDTs have poor field stability and sensitivity compared to HRP2 based RDTs [3, 6]. The threat becomes real in view of the big volumes of HRP2 RDTs required for *P. falciparum* parasite confirmation in Africa and the limited options available of WHO approved non-HRP malaria RDTs [2, 3, 6, 7]. It is, therefore important that decisions to change *pfhrp2* RDTs are based on quality data generated from well conducted studies using recommended methods to avoid unnecessary costly switch of RDTs [6]. However, the designs and methodologies used to investigate, confirm and report *pfhrp2/3* gene deletion studies in Africa have varied. There have been variations in; (1) the size of the studies, (2) source of participants used (health facility versus survey data), (3) clinical classifications of the participants including symptomatic versus asymptomatic individuals, and (4) investigation of *pfhrp2* deletion alone versus *pfhrp2* and *pfhrp3* double deletions and flanking genes and (5) the laboratory methods.

Due to this variability in study designs, methodologies and reporting, the WHO Global Malaria Programme published a standard protocol on the recommended approaches and methods required for investigation, confirmation and reporting of *pfhrp2* and *pfhrp3* gene deletion [7]. This review aims to assess the current status of *pfhrp2* gene deletion and the methods and approaches being used for its estimation, confirmation and reporting in Africa.

## Methods

### Review question

The review aimed to (1) assess the status of *pfhrp2* gene deletion in *P. falciparum* parasites in Africa since 2010 when the first deleted parasites were identified in clinical samples in the Amazon region, (2) assess the methodologies and approaches being used for *pfhrp2/3* gene deletion estimation, confirmation and reporting in Africa.

### Search strategy

A systematic search of literature was conducted electronically for published studies on *pfhrp2*/*3* gene deletion in Africa between January 2010 and June 2019. Literature search was done using PubMed and MEDLINE google Scholar for all articles published in English about *pfhrp2/3* gene deletion in Africa. The following were used as search words; ‘Malaria’, ‘*Plasmodium falciparum’*, ‘*pfhrp2*’, ‘*pfhrp3*’ ‘Gene deletion’, ‘Malaria Rapid diagnostic tests’, ‘Africa’. All searches were restricted to paper titles and abstracts.

### Review period and selection criteria of articles

The review considered the period from January 2010, when Gamboa et al. first reported the occurrence of *pfhrp2/3* gene deletion in clinical samples in Peru until June 2019 [4]. The articles were selected based on the following selection criteria: (1) Original publication, (2) containing primary data on *pfhrp2* deletion, (3) conducted in Africa, and (4) published during the selected review period. In order to expand on the scope, the papers referenced or cited in the selected papers were also reviewed for additional evidence. The WHO recommended protocol for investigation, confirmation and reporting of *pfhrp2/3* gene deletion (Table 1) was used to assess the designs, approaches and methodologies used in the selected relevant articles [7].

**Table 1:** WHO protocol and recommended methods for investigating *pfhrp2* gene deletion [7]

Area of assessment	WHO protocol recommendation
Study design	Cross-sectional (survey)
Participants	Symptomatic with fever (axillary temperature of > 37.5 °C)
Study sites	Distribution of sites in wide range of epidemiological settings; low, moderate and high transmission should be considered
Size of the study	At least 370 individuals enrolled from 10 randomly selected facilities per survey domain or region is recommended
Double deletion (*pfhrp2* and *pfhrp3*) versus *pfhrp2* Only	Protocol recommends investigation of *pfhrp2* and *pfhrp3* double deletions as opposed to *pfhrp2* gene deletion alone
Flanking genes	There is no restriction on the inclusion of the upstream and downstream flanking genes. It remains optional only for characterization of sub-telomeric deletions
Minimum Laboratory Methods Required for confirmation of *pfhrp2/3* gene deletions	Suspected Sample should be HRP2 RDT− and Microscopy+ or HRP2 RDT− and Pf-pLDH RDT+ (only discordant samples should be suspected), Confirmation of *P. falciparum* infection by DNA PCR, demonstration of absence of *pfhrp2/3* genes by gene specific PCR and demonstration of presence of single copy genes MSP1 and MSP2

The status of gene deletion reported and the methods used for its investigations in every searched article were assessed and summarized based on the WHO protocol recommendation for investigation and confirmation of *pfhrp2* and *pfhrp3* gene deletions in *P. falciparum.* Searched material was excluded from the review if they were responses or correspondences to the editor, if they were conference presentations, if year of data collection was outside the review period and where retrieve full text for review was not possible.

In order to standardize methods for estimation and reporting of *pfhrp2/3* gene deletion, the WHO Global Malaria Programme developed and published a *pfhrp2/pfhrp3* gene deletion protocol [7].

The aim of the protocol is to provide guidance to countries on the recommended standard and harmonized methods required for confirmation and reporting of suspected *pfhrp2/3* gene deletions in *P. falciparum*. In this systematic review, published papers were assessed against the WHO standard criteria (Table 1).

## Results

**Fig. 1:**
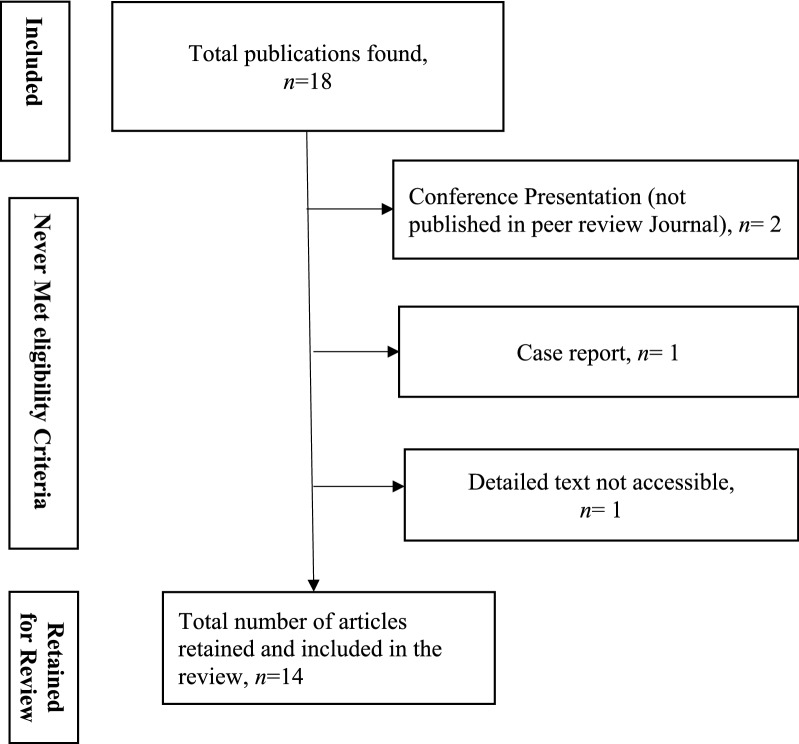
Categorization of the published articles identified in the search that fulfilled the criteria

The review considered published articles on *pfhrp2/3* gene deletion in Africa between Jan 2010 and June 2019 that satisfied our inclusion criteria. The initial search yielded 18 articles however only 14 (77.7%) fulfilled the criteria for inclusion and were retained for review (Fig. 1).

**Fig. 2:**
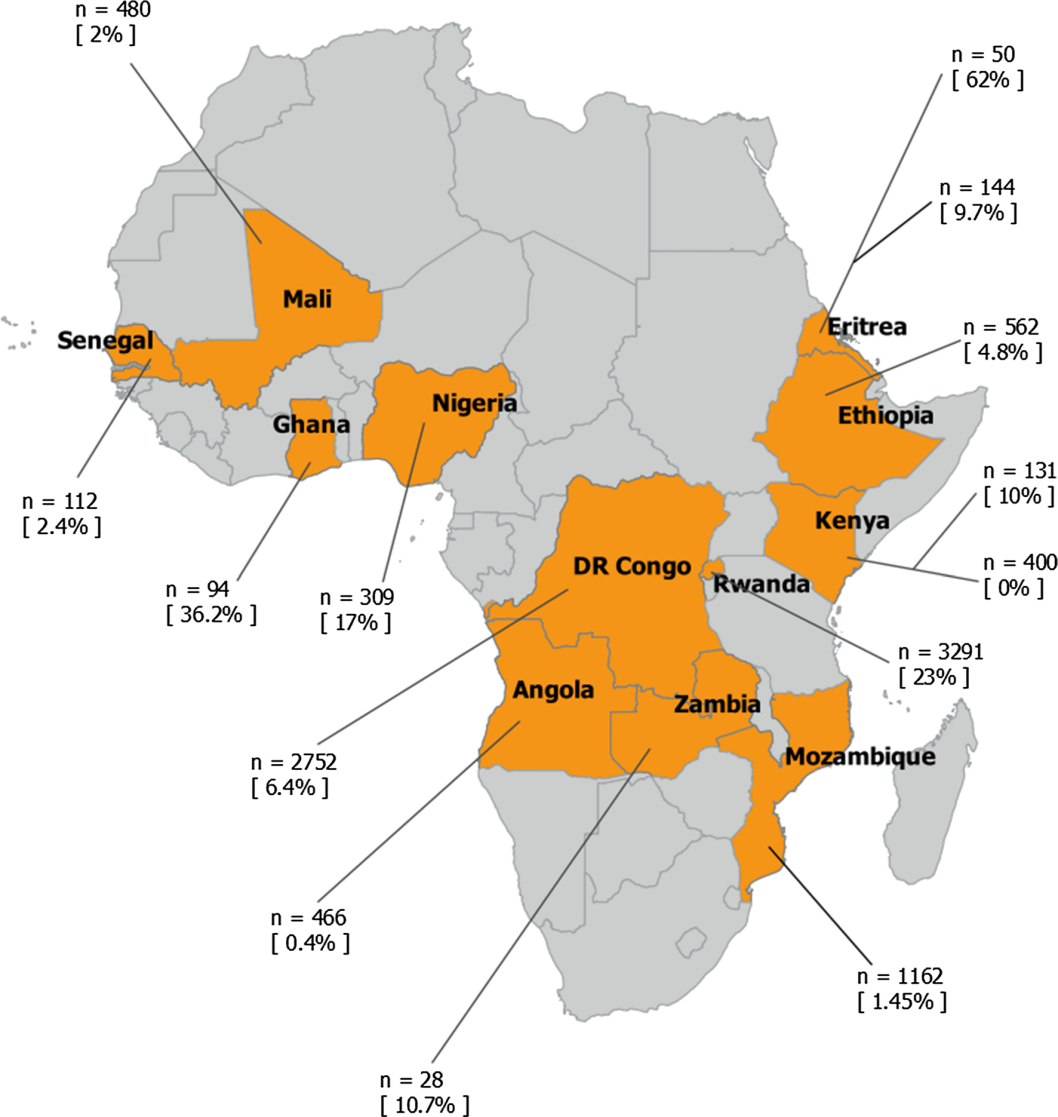
Distribution of reviewed *pfhrp2/3* gene deletion studies across Africa

The summary of findings from the reviewed articles on *P. falciparum pfhrp2* gene deletion based on the WHO recommended methods for confirmation and reporting of *pfhrp2* and *pfhrp3* gene deletions is shown in Table 2. The distribution of reviewed studies of *pfhrp2/3* gene deletion across Africa is shown in Fig. 2.

**Table 2:** Summary of findings from the reviewed articles on *P. falciparum pfhrp2* gene deletion

Country	First author	Areas of assessment					Reported level of *pfhrp2* deletion (%)
		Design and participants	Size of studies (no. of samples)	*pfhrp2* and *pfhrp3* double or *pfhrp2* single deletion	Flanking genes	Lab methods	
Mali [9]	Koita	Cross-sectional, Asymptomatic individuals	480	Only *pfhrp2* deletion reported	Not reported	MSP2 PCR not reported	2
Kenya [16, 18]	Beshir	Cross-sectional, asymptomatic	131	Both *pfhrp2* and *pfhrp3* reported	Not reported	All methods reported	10^a^
	Nderu	Cross-sectional, symptomatic individuals	400	Both *pfhrp2* and *pfhrp3* reported	Not reported	Details not accessed for review	0
DRC [15]	Parr	Cross-sectional, Asymptomatic individuals	2752	Both *pfhrp2* and *pfhrp3* reported	Not reported	MSP1 and MSP2 PCR not reported	6.4
Eritrea [10, 11]	Berhane	Cross-sectional, Symptomatic individuals	50	Both *pfhrp2* and *pfhrp3* reported	Reported	All Lab methods reported	62
	Menegon	Cross-sectional	144	Both *pfhrp2* and *pfhrp3* reported	Not reported	Not available for review	9.7
Rwanda [8]	Kozycki	Cross-sectional, Symptomatic individuals	3291	Only *pfhrp2* reported	Not reported	MSP1 and MSP2 PCR not reported	23^a^
Mozambique [12]	Gupta	Cross-sectional, Symptomatic individuals	1162	*pfhrp2* and *pfhrp3* reported	Not reported	MSP1 and MSP2 PCR not reported	1.45^a^
Senegal [14]	Wurtz	Cross-sectional, Symptomatic individuals	112	*pfhrp2* and *pfhrp3* reported	Not reported	MSP1 and MSP2 PCR not reported	2.4
Ghana [17]	Amoah	Cross-sectional, asymptomatic individuals	94	Only *pfhrp2* reported	Not reported	MSP1 and MSP2 PCR not reported	36.2
Nigeria [13]	Funwei	Prospective cohort	309	*pfhrp2* and *pfhrp3* reported	Not reported	All Lab methods reported	17^a^
Zambia [19]	Kobayashi	Cross-sectional, asymptomatic	28	*pfhrp2* and *pfhrp3* reported	Not reported	MSP1, MSP2 not reported	10.7^a^
Angola [20]	Plucinski	Cross-sectional, asymptomatic	466	*pfhrp2* and *pfhrp3* reported	Not reported	MSP1, MSP2 not reported	0.4
Ethiopia [21]	Girma	Cross-sectional, asymptomatic	562	*Only pfhrp2 reported*	Not reported	Not available for review	4.8^a^

^a^*pfhrp2* gene deletion estimate was based on a smaller denominator rather than total *P. falciparum* infected samples

## Discussion

The status, methods and approaches that have been used for confirmation and reporting of *pfhrp2*/*3* gene deletions in Africa were assessed and reviewed where studies were conducted and reported between 2010 and June 2019. There was wide variation in methods and approaches used across studies (Table 2), as compared to those recommended in the WHO standard protocol for confirmation and reporting of *pfhrp2* gene deletions (Table 1). Studies varied from the designs, size and sampling, whether they assessed both *pfhrp2* and *pfhrp3* double deletions or *pfhrp2* single deletion alone with wide variation in laboratory methods.

### Summary of results of studies on *pfhrp2* gene deletion

A total of 14 research articles satisfied our criteria for inclusion in the review (Table 2). These articles provide unequivocal evidence of the existence and occurrence of *pfhrp2* and *pfhrp3* gene deletion in Africa where *P. falciparum* is the predominant parasite and where huge volumes of HRP2 based RDTs are used for malaria diagnosis [8–18]. Based on the articles included under this review, the current levels of *pfhrp2/3* gene deletion across malaria endemic countries in Africa range from the highest 62% in Eritrea to the lowest 1.45% and 0.4% in Mozambique and Angola, respectively [11, 12, 20]. However, levels of gene deletions were as high as 80% at some hospitals in Eritrea [11]. Gene deletions were not detected in one of the studies in Kenya [16]. The observed differences in the levels of *pfhrp2/3*gene deletion in these studies could be due to selection pressure caused by exclusive use of HRP2 RDTs over time as suggested in previous studies [6, 11]. In Eritrea, HRP2-based RDTs had been widely used exclusively since 2006 and that provided ideal conditions for selection and spontaneous occurrence of *pfhrp2*/*3* negative parasites that remained undetected and continued to increase [11]. This selection pressure and spontaneous occurrence of gene-deleted parasites has been predicted by recent mathematical modelling, that showed that exclusive use of HRP2-based RDTs exerts strong selection pressure for *pfhrp2/3*-negative parasite populations leading to their increase in frequency [22]. Low malaria transmission setting in Eritrea could have also contributed to selection of *pfhrp2/3*-negative parasites once they emerge [6, 11]. The extremely low prevalence of *pfhrp2/3* deleted parasites reported by studies in Western Kenya and Southern Mozambique may be due to the absence of these ideal conditions for *pfhrp2* selective pressure, such as are high transmission settings and use of malaria microscopy as the major diagnostic tool [12, 16]. Due to the high levels of *pfhrp2*/*3* gene deletions above the 5% recommended WHO cut-off, Eritrea has introduced non-HRP2 RDTs to detect *pfhrp2* and *pfhrp3* deleted parasites [11].

### Limitations of the studies and how they affect the results

There was wide variation in the approaches and methods used for investigation, confirmation and reporting of *pfhrp2* and *pfhrp3* gene deletion across all studies. The major differences and limitations in methods and approaches across the studies are highlighted below.

#### Study designs and participants

All the reviewed studies but one used a cross-sectional design as recommended by the WHO protocol for confirmation and reporting *pfhrp2* and *pfhrp3* gene deletion [8–18]. The WHO protocol recommends recruitment and enrolment of febrile symptomatic participants seeking treatment at health facility into *pfhrp2* and *pfhrp3* deletion studies [7]. The reason for preference of symptomatic to asymptomatic population for *pfhrp2/3* gene deletion studies is because parasite density is generally higher in the former compared to the low-density infections in the latter and hence provide better quality samples for confirmation of gene deletions by molecular tests [6, 7]. However, a number of studies included in the current review collected and investigated samples from asymptomatic individuals for *pfhrp2*/*3* gene deletion investigation including blood donors in Mali [9]. The DRC, Ethiopia and Mozambique studies estimated gene deletion in samples collected from Demographic Health Survey (DHS) and population-based surveys in asymptomatic individuals reporting the levels of *pfhrp2* gene deletions as 6.4%, 4.8% and 1.45%, respectively [12, 16, 21]. These levels of deletions are relatively lower compared to the Eritrea and the Rwanda studies that recruited febrile symptomatic individuals and reported very high levels of *pfhrp2* gene deletions up to 62% and 23%, respectively [8, 11]. The WHO standard protocol recommends symptomatic individuals as the preferred study participants for *pfhrp2*/*3* gene deletion studies. The Mali study however gives contrary findings and reports a significant association between asymptomatic population and *pfhrp2/3* gene deletion [9]. These variations in methodologies call for standardization of methods, approaches and reporting of *pfhrp2*/*3* gene deletion studies across malaria endemic countries in Africa as the use of appropriate study participants is fundamental for the gene deletion study outcomes.

#### Size of the studies

The WHO protocol for investigation of *pfhrp2/3* deletion recommends the recruitment of a minimum of 370 or 318 symptomatic individuals with suspected *P. falciparum* infection to estimate a prevalence of 3.2% and 8.0%, respectively per sampling region or province [7]. However, the articles included under this review showed wide variation in size with regard to number of participants recruited. In some cases, extremely low sample sizes were used such as the Eritrean study that enrolled a total sample of 51 individuals to report a *pfhrp2* gene deletion of > 80% at one of the study hospitals [11]. The Zambian study reported a gene deletion of up to 10.7% (3/28) based on a total of 28 *P. falciparum* DNA samples [19]. Based on the WHO protocol, even when the recommended sample is used, it should be distributed and spread across all regions to provide a representation of a country’s malaria epidemiology and *P. falciparum* population [7]. However, these criteria on sample size and its distribution across different malaria epidemiological setting were not complied with in a number of articles reviewed. The effect and challenges associated with the use of inadequate sample size on study outcomes in prevalence studies are widely published [23, 24]. Non-uniformity and non-compliance to the recommended methods may pose challenges in reporting and comparability of findings on *pfhrp2/3* gene deletion across countries.

Reporting of *pfhrp2* and *pfhrp3* double deletion versus *pfhrp2* single deletion alone: The WHO protocol for *pfhrp2/3* studies recommends estimation and reporting of both *pfhrp2* and *pfhrp3* in *P. falciparum* gene deletion studies [3, 6, 7]. This is because *P. falciparum*-based RDTs are designed to specifically recognize HRP2 antigen, however *pfhrp2* and *pfhrp3* are homologous genes whose antigens may cross-react due to high degree of similarity in their amino acid sequence [5, 25, 26]. A number of studies elsewhere have reported a possible association between *pfhrp2* and *pfhrp3* gene deletions warranting investigation of both genes. Evidence from the Eritrean study showed that every sample that was *pfhrp2* deleted was also *pfhrp3* deleted suggesting a possible association [11]. However, the studies considered under this review exhibited variation in approaches with some investigating and reporting *pfhrp2* and *pfhrp3* double deletion while others reported *pfhrp2* deletion alone. Gene deletions studies in Ghana, Mali and Rwanda investigated and reported single *pfhrp2* deletion alone [8, 9, 17], while those conducted in DRC, Mozambique, Kenya, Eritrea and Nigeria investigated and reported *pfhrp2* and *pfhrp3* double deletions [11–16, 18]. The effect of investigating single *pfhrp2* gene deletion alone is a possible underestimation as some of the samples may test positive with HRP2 RDT even when parasites are *pfhrp2* deleted due to cross-reactivity with HRP3 antibodies. These observations and variations in methods call for harmonization and standardization of investigative and reporting approaches for *pfhrp2/3* gene deletions.

#### Laboratory methods

The recommended laboratory-based testing methods required for confirmation of suspected *pfhrp2*/*3* gene deletion in *P. falciparum* parasites have been previously published [4, 6, 25–35]. At the minimum, the suspected sample for *pfhrp2/3* deletion should be negative by HRP2 based RDT and positive with expert microscopy or Pf-pLDH RDT [6, 33, 35]. From a suspected deleted sample, a dried blood spot is collected for PCR to confirm *P. falciparum* mono-infection and exclude other non-*P. falciparum* species. Samples that are PCR confirmed as *P. falciparum* are amplified in the exon1 and exon 2 regions of the *pfhrp2/3* gene to detect the presence or absence of the gene [25, 33–35]. Samples that fail to amplify *pfhrp2* or *pfhrp3* in the exon region are considered *pfhrp2* and *pfhrp3* deleted after ascertaining the quality of parasite DNA by amplification of MSP1 and MSP1 single copy genes [4, 6, 7]. However, not all articles considered under this review performed the minimum recommended laboratory testing required for confirmation of parasite gene deletion. The study in Ghana extracted and used blood sample left-overs from used RDT test cassettes for *pfhrp2/3* gene deletion study as opposed to the use of dried blood spots as preferred samples [17]. The effect of using wrong samples on the final *pfhrp2/3* gene deletion outcome is poorly understood. Four of the reviewed articles that reported *pfhrp2* deletion in four countries missed an essential procedural requirement of demonstrating the quality of parasite DNA by PCR amplification of MSP1 and MSP2 single copy genes of *P. falciparum* [8, 14, 15, 17]. One study reported MSP1 alone and the reason for not amplifying MSP2 was not indicated [9]. Detailed laboratory methods for one of the articles could not be accessed [16]. Failure to demonstrate the presence of *P. falciparum* MSP1 and MSP1 single copy genes as an essential confirmation of *P. falciparum* DNA quality in suspected *pfhrp2/3* deleted samples is a major methodological flaw that creates uncertainty on the validity and correctness of the reported deletion estimates [6, 7]. The investigation of deletions in flanking genes located upstream and downstream of *pfhrp2/3* in the subtelomeric region is optional and not essential requirement for confirmation and reporting of *P. falciparum* parasite gene deletion [6, 7]. However whole genome sequencing studies have showed that deletion is not restricted in the *pfhrp2* and *pfhrp3* gene regions and can extend in the neighbouring flanking genes [6, 36]. Under the current review, all the articles except one did not investigate or report deletions in the flanking genes.

#### Spread and distribution of study sites

The WHO recommends the design of *pfhrp2/3* surveys that aims to achieve representativeness of a country’s malaria parasites population across all epidemiological settings [7]. The importance of spreading the sample across the country to achieve geographical representation is emphasized in the Indian and DRC studies that showed a wide variation in frequency and occurrence of *pfhrp2* and *pfhrp3* gene deletion in parasites collected across the various states and provinces [15, 31]. However, under the current review, apart from one study that used a national representative sample of parasites collected under the Health Demographic Survey (DHS), the rest of the reported studies had relatively limited geographical coverage that may not be representative of the entire country’s *P. falciparum* parasites population [8–14, 16–18]. The direct effect of this methodological approach is the difficulty it presents in determining the correct estimate and extent of spread of parasite gene deletion that is representative of a country’s parasite population.

#### Denominators used for computation of *pfhrp2* gene deletion estimates

Across all the reviewed studies, there were differences in the denominators used in the final computation of gene deletion estimates. While others used total *P. falciparum* infections as measured by microscopy, others used PCR confirmed or number of RDT-/microscopy + discordant samples that is a smaller denominator [8–18]. The use of these different denominators leads to different *pfhrp2/3* gene deletion estimates with possible overestimation or underestimation. The WHO standard protocol recommends the use of total *P. falciparum* infections measured by microscopy the suitable denominator to avoid overestimation of gene deletion estimates [7]. However, this has limitations for erroneous inclusion of non-*P. falciparum* species and false positives that are misclassified by poor quality microscopy. This potentially inflates the denominator leading to under estimation of deletions [6, 12, 13].

### Implications for future research and future perspectives

Our review found a wide variation in methodologies and approaches for investigation of *pfhrp2*/*3* gene deletion across studies in malaria endemic countries in Africa. The direct implication of the use of non-Standardised and non-harmonized methods for confirmation and reporting of parasite gene deletion is the risk of unnecessary recommendations for a costly switch from HRP2 based RDTs to non-*P. falciparum* RDTs. Non-HRP2 RDTs are more expensive, less sensitive with poor field thermal stability [3, 6]. Unnecessary switch of current diagnostic strategies may potentially undermine the current gains and improvement in parasite-based diagnosis especially in Africa where *P. falciparum* is predominant and where large volumes of HRP2 based RDTs are used for malaria diagnosis [1, 2]. However, future research could consider establishment of the actual costs associated with the process of switching diagnostic tools and the public health benefit of deploying non-*P. falciparum* RDTs in the context of gene deletions.

Despite the high burden and dominance of *P. falciparum,* the search identified only 13 published articles on *pfhrp2/3* gene deletion in Africa. This observation could explain the limited data available on the occurrence and status of *pfhrp2*/*3* gene deletion in malaria endemic countries in Africa. The WHO recommends initiation of surveys and surveillance systems to allow early detection and containment of *pfhrp2*/*3* gene-deleted parasites in countries at risk of this threat [3, 7]. Specifically the high risk countries are those located in regions where gene deletions have been confirmed, where there are concerns of false negative RDTs results and where discordance rate between microscopy and RDT is high [3, 7]. However, the direct implication for continued use of HRP2 RDTs in countries at risk without deliberate surveillance systems to allow early detection of gene-deleted parasites is a potential risk for selection pressure and continued spread of these parasites [3, 11, 37]. Even when there is initial confirmed presence of *pfhrp2/3* gene deletion, the WHO recommends the need for periodic monitoring to assess if levels are increasing or exceeded the 5% prevalence cut-off required for change of diagnostic policies [7]. However, future research could consider generating additional evidence on the actual contribution of gene-deleted parasites to transmission, malaria morbidity and mortality if left to spread undetected.

Previous studies have demonstrated the possible occurrence and survival of *pfhrp2* and *pfhrp3* gene-deleted parasites in all malaria epidemiological setting including low and high transmission zones [9, 11, 15, 31]. However, the extent to which these undetected and unreported gene-deleted parasites affect surveillance and disease burden estimates is glaringly missing and remains subject of further research. From the review, there is evidence of the ability of *pfhrp2*/*3* gene-deleted parasites to spread and cause disease [6, 9, 11]. However, their role and actual contribution in causing severe disease and deaths needs to be studied further. There are key questions on whether *pfhrp2/3* deleted parasites are drug sensitive compared to gene harbouring parasites and whether current treatment is effective for *pfhrp2*/*3* deleted parasites.

Studies have shown the failure of HRP2 based and the ability of non-HRP2 RDTs to detect *pfhrp2/3* gene-deleted parasites in *P. falciparum* infected samples [6, 11, 12]. Indeed, the advance in the development of robust diagnostic tools to detect gene-deleted parasites is enormous [4, 21, 25, 32–35, 38, 39]. In addition to the current molecular and serological tools, Plucinski et al. have developed a bead-based multiplex assay that simultaneously detects parasite aldolase, parasite lactate dehydrogenase and histidine rich protein 2 increasing the possibility of detecting gene-deleted parasites [20]. However, false deletions due to unamplified *pfhrp2/3* could still occur due to low quality parasite DNA particularly in low parasitaemia samples. Missed deletions could occur if an infection with a deleted parasite occurs subsequent to an infection with a wild type parasite, since circulating HRP2 can persist for up to a month [6]. In high transmission settings, such as many parts of Africa, polyclonal infection that involves host co-infection with two or more parasite strains is common. Co-infection may involve a non-deleted strain masking a gene-deleted strain that presents a challenge for the current diagnostic tools. This calls for future research into more robust diagnostic tools to detect masked gene-deleted parasites.

## Conclusion

Based on the review, there is evidence of the presence of *pfhrp2/3* gene-deleted *P. falciparum* parasites in Africa. The approaches and methods used for investigation, confirmation and reporting of *pfhrp2/3* deleted parasites have varied between studies and across countries. The available evidence on the occurrence of *pfhrp2/3* deletion comes from a limited number of countries leaving it largely unknown and unreported in many malaria endemic countries in Africa. Countries that are considering plans to confirm and report *pfhrp2/3* deletion should use recommended standard and harmonized methods to prevent unnecessary recommendations for costly switch of RDTs in Africa.

## Data Availability

Not applicable.

## Abbreviations

HRP2: histidine rich protein 2
*pfhrp2*: *Plasmodium falciparum* histidine rich protein 2
RDTs: rapid diagnostic tests
ACT: artemisinin-based combination therapy
MSP1: merozoite surface antigen 1
MSP2: merozoite surface antigen 2
PCR: polymerase chain reaction
WHO: World Health Organization
ITNs: insecticide-treated mosquito nets
LLINs: long-lasting insecticide-treated nets
IRS: indoor residual spraying
